# The Impact of Coding Levels of Magnitude and of Spatial-Direction on the Spatial–Numerical Association of Response Codes Effect of Negative Numbers

**DOI:** 10.3389/fpsyg.2022.865003

**Published:** 2022-05-30

**Authors:** Xiaojin Zeng, Jian Zhang, Longnong Dai, Yun Pan

**Affiliations:** ^1^School of Physical Education, Guizhou Normal University, Guiyang, China; ^2^School of Psychology, Guizhou Normal University, Guiyang, China

**Keywords:** coding levels, magnitude, negative numbers, spatial-direction, SNARC effect

## Abstract

Whether negative numbers have a fixed spatial–numerical association of response codes effect (SNARC effect), and (if they have) whether the spatial representation of negative numbers is associated with negative numbers’ absolute or signed values remains controversial. In this study, through three experiments, the coding level of the magnitude and the spatial-direction is manipulated. In the first experiment, participants are required to code the magnitude and spatial-direction explicitly by using a magnitude classification task. In the second experiment, participants are forced to code the magnitude implicitly as well as to code the spatial-direction explicitly by utilizing a cuing task. In the third experiment, participants are obliged to code the magnitude explicitly as well as to code the spatial-direction implicitly by adopting a magnitude and arrow-direction classification tasks with Go/No-Go responses. The results show that (1) the absolute value of negative numbers associates with space when the magnitude of negative numbers is explicitly coded, no matter employing the explicit or implicit spatial-direction; (2) the signed value of negative numbers associates with space under the condition of implicit magnitude as well as explicit spatial-direction. In conclusion, the current study indicates that the SNARC effect of negative numbers is variable in different conditions, and the type of SNARC effect about negative numbers is modulated by the joint coding level of the magnitude and spatial-direction.

## Introduction

Numbers provide a foundation for learning to understand the world. Therefore, it is important to determine how we mentally represent numbers. In the past two decades, the association between numbers and space, known as spatial–numerical associations (SNAs), was revealed by a series of representative reports. It was initially discovered in the positive numbers by Dehaene et al. (1993). In their research, participants performed a parity judgment task, deciding whether a positive number was odd or even by pressing left- and right-located keys. The results showed that participants’ left-hand responses were faster when the stimulus was a small number, while their right-hand responses were faster when the stimulus was large. This is defined as the spatial–numerical association of response codes effect (SNARC effect). Researchers explain this effect with the existence of a left-to-right mental number line (MNL), suggesting that numbers are represented horizontally in individual’s mind from left to right based on their magnitudes on the MNL. Small numbers are represented on the left, and large numbers are represented on the right. Therefore, when participants respond to numbers, small numbers will promote left-hand responses, while large numbers boost right-hand responses. Thereafter, substantial studies started to investigate the SNARC effect with positive numbers by using different tasks. Gevers et al. (2006) adopted a magnitude classification task instructing participants to decide whether a number was larger or smaller than a given standard number by pressing left- and right-located keys. They found positive numbers have a SNARC effect in the task. Fischer et al. (2003) used an altered Posner cuing paradigm to investigate whether positive numbers can automatically attract individuals’ attention to the left or right space. They instructed participants to fix their eyes on the position of positive numbers but to ignore the magnitude. When left-right distributed target stimuli occurred, participants were instructed to point to the position of target stimuli by using left- and right-located keys. In this way, whether the cuing of positive numbers had influences on attentional bias could be clearly investigated. In this condition, they found that small positive number cues facilitated the detection of a target on the left, and large positive number cues facilitated the detection on the right. This phenomenon extended the SNARC effect of positive numbers to the attentional field and was defined as the attentional SNARC effect. Next, Fischer and Shaki (2016) performed a magnitude and picture-direction classification task with Go/No-Go responses to examine whether magnitude of positive numbers and space were still associated when the explicit information of spatial-direction was excluded. In their task, participants were instructed to judge whether the magnitude of positive numbers and the direction of pictures matched with a certain instruction. For example, one of the instructions is responding to numbers smaller than five or left-facing car pictures. If a stimulus matched with a certain instruction, participants were required to respond with the space bar rather than left- and right-located keys. If a stimulus did not match with a certain instruction, participants were instructed to wait until the stimulus disappeared on the screen. Their results were similar to those of previous studies of positive numbers. In other word, they duplicated the SNARC effect, and proved a pure conceptual association between positive numbers and space.

According to Shaki and Fischer (2018), there are two key factors that should be paid attention to when investigating the positive numbers’ SNARC effect. They are magnitude and spatial-direction. In addition, each of the two factors has two coding levels, which are implicit and explicit coding levels. For the magnitude, implicit coding of magnitude “*ensures that any magnitude effect on performance reflects obligatory semantic processing that was not merely instructed by the task*” (p. 109). Explicit coding of magnitude needs direct instructions for participants to focus on magnitudes of numbers in a task. For the spatial-direction, implicit coding of spatial-direction uses “*only a single central response key and (in half of the trials) a single central number*’ to remove ‘*explicitly spatial features during number assessment*” (p. 110). Explicit coding of spatial-direction has bilateral-distributed or left- and right-located response keys, so that maintains explicit spatial features in the responsive phase.

In the perspective of coding levels, it can be found that the coding level of the two factors is different in abovementioned studies of positive numbers’ SNARC effect. In the study of Gevers et al. (2006), both the magnitude and spatial-direction are explicitly coded in this task. Although some researchers argue that the magnitude classification task could be performed based on the order of numbers rather than their magnitude (Pitt and Casasanto, 2020; Mingolo et al., 2021), others believe that participants indeed process the magnitude in the task; at least, the magnitude and order are both relevant in SNARC and SNARC-like effects (Prpic et al., 2021). In other words, some researchers deem that order is explicitly coded in magnitude classification task rather than magnitude, the others take for a more comprehensive standpoint. Here, as one of the mainstream opinions advocated, the magnitude in the task could be considered as being explicitly coded. Thus, both the magnitude and spatial-direction are explicitly coded in this task. In the study of Dehaene et al. (1993) and Fischer et al. (2003), the magnitude is implicitly coded whereas spatial-direction is explicitly coded. In the study of Fischer and Shaki (2016), the magnitude is explicitly coded whereas spatial-direction is implicitly coded. However, no matter what coding levels of magnitude and spatial-direction, there is a link between positive numbers and space. Namely, the SNARC effect of positive numbers can be evoked. Although some researchers doubt the existence of positive numbers’ attentional SNARC effect (Pellegrino et al., 2019; Pellegrino et al., 2021; but Fischer et al., 2020), many researchers still acknowledge that SNAs of positive numbers exist when magnitude and spatial-direction codes are jointly used (Gertner et al., 2013; Antoine and Gevers, 2016; Kopiske et al., 2016; Dixon, 2017; Cleland and Bull, 2019; Pinto et al., 2019; Aleotti et al., 2020), unless both magnitude and spatial-direction are implicitly coded (Shaki and Fischer, 2018).

While evidences for SNARC effect in positive numbers are very consistent, previous studies for SNARC effect in negative numbers have yielded inconsistent results. Shaki and Petrusic (2005) conducted a magnitude comparison task under two conditions. The first condition had stimuli of mixed positive and negative number pairs. In this condition, smaller numbers coincided with the negative ones, and larger numbers coincided with the positive ones. The second condition had stimuli of pure negative number pairs. Participants were instructed to compare the magnitude of number pairs by using left- and right-located keys. They found that left-hand responses were faster for a smaller number, while right-hand responses were faster for a larger number when mixed number pairs were compared. This indicated a SNARC effect and proved the space was associated with signed values of negative numbers. However, they also found left-hand responses were faster for a larger number, while right-hand responses were faster for a smaller number when pure negative number pairs were compared. This implied a reversed SNARC effect and demonstrated an association between the absolute value of negative numbers and space. Conversely, Nuerk et al. (2004) conducted a parity judgment task with pure negative number pairs and didn’t discover any stable standard or reversed SNARC effect. This forecasted that negative numbers and space did not have associations. Fischer and Roitmann (2005) improved the study of Nuerk et al. (2004) by manipulating participants’ experiences of numbers. They found similar results that negative numbers did not automatically elicit SNAs in the parity judgment task. Dodd (2011) adopted the cuing task to explore whether negative numbers determined the attentional SNARC effect. They found that “*the presentation of negative numbers did not lead to a reversal of the standard attentional SNARC effect, but it did lead to the elimination of the effect*” (p. 7). Fischer and Shaki (2017) performed a magnitude and picture-direction classification task with Go/No-Go responses to investigate the SNARC effect of negative numbers. They found a SNARC effect for left-to-right counters, indicating an association between the signed value of negative numbers and space. However, they also found that for right-to-left counters, stable SNAs were disappeared.

Considering these abovementioned studies of negative numbers, whether negative numbers have a fixed SNARC effect (or reversed SNARC effect) and whether space associates with negative numbers’ absolute or signed values remains controversial. However, it can be found that the main difference of these studies is the coding level of the magnitude and spatial-direction. These different coding levels in different tasks may lead to various spatial representation of negative numbers. Specifically, it is reasonable to pay attention to the impact of coding levels of magnitude on negative numbers’ SNARC effect, because the SNARC effect is an effect about the coding relationship between the magnitude and space (according to one of the mainstream opinions). Meanwhile, since the SNARC effect occurs in the response-selection related stage (Nan et al., 2021; Yan et al., 2021) and spatial-direction is a key factor in this stage (Shaki and Fischer, 2018), it is rational to imagine that the coding level of spatial-direction has a potential effect on negative numbers’ SNARC effect. To sum up, we assume that the joint coding level of the magnitude and spatial-direction is a modulating factor of spatial representation of negative numbers.

In the current study, we intend to choose some classical studies of negative numbers’ SNARC effect to replicate. These replication studies should include various combinations of coding levels. Thus, we can investigate the spatial representation of negative numbers under the condition of different combinations of coding levels. And in this way, we can examine whether the joint coding level is a modulating factor and provide a whole picture of spatial representation of negative numbers.

According to the definition of Shaki and Fischer (2018), a magnitude comparison task in the study of Shaki and Petrusic (2005) is chosen for our first experiment to investigate the condition of explicit magnitude and spatial-direction coding; a cuing task in the study of Dodd (2011) is adopted for our second experiment to examine the condition of implicit magnitude coding and explicit spatial-direction coding; and the magnitude and picture-direction classification tasks with Go/No-Go responses in the study of Fischer and Shaki (2017) is used for our third experiment to explore the condition of explicit magnitude coding and implicit spatial-direction coding (see Table 1). Apart from these experiments, as far as we know, there is no study investigating the spatial representation of negative numbers under the condition of implicit coding of magnitude as well as spatial-direction. However, Shaki and Fischer (2018) have proved that positive numbers and space do not have an association if magnitude and spatial-direction are all implicitly coded. Therefore, this scenario is excluded from our study. Ultimately, our study includes three experiments. Every experiment is corresponding to a replication study. Consequently, we can investigate the impact of the coding level of magnitude and spatial-direction on the spatial representation of negative numbers.

**TABLE 1:** Summary of results for each combination of coding levels in different classical studies (for details, see text).

Magnitude		Explicit	Implicit
Spatial-direction	Explicit	See in study of Shaki and Petrusic (2005): • A reversed SNARC effect exists. The absolute value of negative numbers is associated with space.	See in study of Dodd (2011) • An attentional SNARC effect does not exist.
	Implicit	See in study of Fischer and Shaki (2017): • A SNARC effect exists for individuals with a left- to right- counting habit. The signed value of negative numbers is associated with space.	• None

In the processing of conducting these replication studies, some potential methodological defects in previous studies have been improved. In this way, our hypothesis can be more accurately investigated. Specifically, in the first experiment, we improved the experiment of Shaki and Petrusic (2005) by removing number zero and adopting a magnitude comparison task with stimuli of pure negative numbers. In their study, they used number zero as a stimulus and compared number zero with negative numbers. However, according to the study of Varma and Schwartz (2011), number zero can lead to a change of some magnitude effects, such as the size effect and distance effect. At the same time, Wood et al. (2006) proposed that “*the number 0 alone was responsible for the significant SNARC slope for the Arabic number notation with the interval 0 to 9*” (p. 1071). Thus, it is possible that the number zero can influence the SNARC effect of negative numbers. To avoid some latent influences of number zero on the spatial representation of negative numbers, abovementioned improvements are adopted. In the second experiment, we improved the first experiment of Dodd (2011) by increasing the time of cue from 300 to 500 ms to ensure that participants had more time to implicitly code the magnitude. In replication studies of attentional SNARC effect of positive numbers, some researchers adopted 300 ms as the time of cue (Pellegrino et al., 2019), others chose 500 ms as the time of cue (Pellegrino et al., 2021). None of them replicates the attentional SNARC effect found by Fischer et al. (2003). However, in replication studies of attentional SNARC effect of negative numbers, except for the study of Dodd (2011) which uses 300 ms as the time of cue, there is no other study investigating attentional SNARC effect of negative numbers under a condition of 500 ms or longer cuing time. Considering that the attentional SANRC effect does not exist in Dodd’s (2011) study, we suppose that the following two reasons may account for the inexistence. First, the attentional SNARC effect does not exist for negative numbers. Second, the time of cue is too short for participants to implicitly code the magnitudes of negative numbers. Therefore, to investigate whether longer cuing time determines a stable attentional SANRC effect of negative numbers, we increased the cuing time to 500 ms just as researchers did in previous studies of positive numbers. In the third experiment, we improved the experiment of Fischer and Shaki (2017) by adopting more abstract stimuli of arrow symbols. In the study of Fischer and Shaki (2017), researchers used clipboard pictures of left-facing (or right-facing) cars as stimuli, which included mixed directivity information. When participants, who have a driving license of the Commonwealth and of Japan, respond to car pictures. They often automatically reflect the driving rule of driving along the left side of the road. However, participants, who have a driving license in America or China, often automatically reflect the driving rule of driving along the right side of the road. These left-side or right-side driving rules are distinct in different countries (Shan et al., 2017). When participants respond to the facing direction of car pictures, mixed directivity information from driving rules may potentially distract participants in the task and lead participants to make a wrong decision. Therefore, in our third experiment, car pictures were replaced with arrow symbols – a more abstract symbol about directivity. Thus, in our third experiment, we eliminate the interference of the mixed directivity information contained in driving rules.

According to results in explicit magnitude studies (Shaki and Petrusic, 2005; Fischer and Shaki, 2017), we hypothesize that a reversed SNARC effect would emerge in the first and third experiments. Namely, the absolute value of negative numbers associates with space due to the explicit coding of magnitude despite the coding level of the spatial-direction. However, according to results in implicit magnitude studies (Fischer, 2003; Nuerk et al., 2004; Dodd, 2011), we assume that there are two possibilities about the spatial representation of negative numbers in the second experiment. First, there may be a standard attentional SNARC effect. In other word, the spatial representation of negative numbers would be based on their signed values because participants have enough time to implicitly code magnitude as well as to explicitly code spatial-direction. Second, there may be no standard or reversed attentional SNARC effect for negative numbers. Namely, negative numbers don’t have SNAs under the condition of implicit magnitude coding as well as explicit spatial-direction coding. In summary, we hypothesize that negative numbers have flexible SNAs, and the joint coding level of the magnitude and spatial-direction plays a key role in spatial representation of negative numbers.

## Experiment 1

### Methods

#### Participants

Thirty-two right-handed undergraduate students (15 males, 17 females; mean age = 20.17 years, SD = 1.18) voluntarily participated in the experiment. All participants had normal or corrected-to-normal vision and were unaware of the purpose of the experiment. After a task of counting dots drawn on an A4 paper, we ensured that all participants have a left-to-right counting habit. All the present studies received approval from the relevant ethics committee.

#### Stimuli and Apparatus

The experiment was performed by using E-Prime 2.0 Professional Software (Psychology Software Tools Inc, 2017, Pittsburgh, PA, United States). The target stimuli were negative numbers (–1 ∼ –9) excluding –5, presented in black, with 48 size KaiTi font.

#### Procedure

**FIGURE 1:**
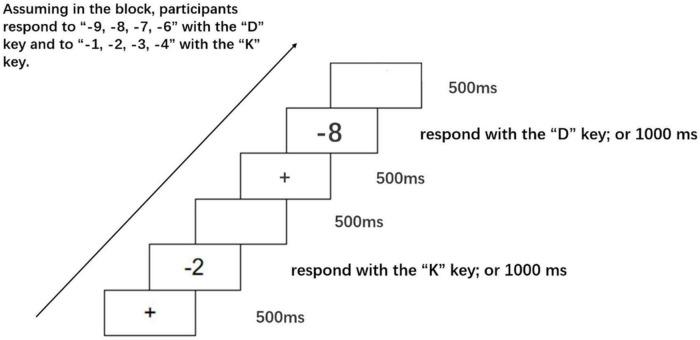
The *processing* of Experiment 1.

In a quiet room, participants were seated before a 15.6-inch Lenovo laptop screen with a resolution of 1366 × 768 pixels. The distance from their eyes to the screen was approximately 57 cm. During the experiment, participants were instructed to respond to target stimuli by pressing the “D” and “K” keys of a standard computer keyboard with their left and right hands, respectively. In each trial, a fixation (“ + ”) was first displayed in the center of the screen with a white background for 500 ms. Next, it was replaced by a negative number (the target stimulus). The target stimulus disappeared after 1000 ms or after a response made by participants. Finally, the trial concluded with a blank screen shown for 500 ms (see Figure 1). During the formal experiment, a total of 320 trials were contained in two blocks (20 repetitions × 8 negative numbers × 2 blocks = 320 trials), in which participants were instructed to judge whether the target number were smaller or larger than –5. In the first block, participants responded to numbers smaller than –5 with their left hand pressing the “D” key, and to numbers larger than –5 with their right hand pressing the “K” key. In the second block, participants responded to numbers larger than –5 with their left hand pressing the “D” key, and to numbers smaller than –5 with their right hand pressing the “K” key. The order of the blocks was counterbalanced across participants. And stimuli of negative numbers were presented randomly. Prior to the formal experiment, a practice session was run, with all target stimuli presented twice.

### Data Analysis

We used a repeated measures ANOVA with magnitude (small “<–5”, large “>–5”), and response hands (left, right) as within-subject factors. The dependent variable was the response time (RT). A significant interaction between the magnitude and response hands would indicate the presence of a SNARC effect or a reversed SNARC effect. To further explore the effect, a regression analysis approach described in previous studies (Lorch and Myers, 1990; Didino et al., 2019) was used. In this approach, the response time difference (dRT), which was equal to the value of the right-hand RT minus the left-hand RT, was computed for each negative number. For each subject, these values were entered into a regression analysis with a negative number as a predictor. A one-sample *t*-test was performed to evaluate whether the mean regression coefficients of the group deviated significantly from zero. A significant *t*-value would suggest a significant SNARC effect or a significant reversed SNARC effect. Specifically, if the mean regression coefficient was negative and the difference from zero was significant, this would indicate a standard SNARC effect. However, if the mean regression coefficient was positive and the difference from zero was significant, this would suggest a reversed SNARC effect.

### Results

**FIGURE 2:**
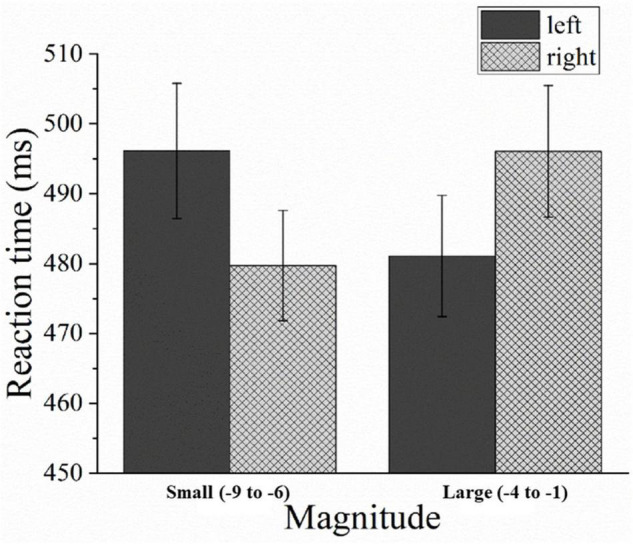
Mean response times (RT) in Experiment 1 as a function of the magnitudes and response hands. Error bars represent 1 standard error of the mean (SEM).

The mean error rate was 6%, and trials with errors were excluded from the analysis. The mean RT for correct trials for both hands was 488 ms (*SD* = 111 ms). The repeated measures ANOVA revealed that there was no significant main effect for the magnitude [*F*(1,31) = 0.048, *p* > 0.05, η*^2^_*p*_* = 0.002] and response hands [*F*(1,31) = 0.064, *p* > 0.05, η*^2^_*p*_* = 0.002]. However, a significant interaction was observed between the magnitudes and response hands [*F*(1,31) = 8.699, *p* < 0.05, η*^2^_*p*_* = 0.219]. This interaction demonstrated a reversed SNARC effect due to faster left-hand (481 ms) than right-hand (496 ms) responses for larger numbers (e.g., –1 and –2), [*F*(1,31) = 5.644, *p* < 0.05]; and faster right-hand (480 ms) than left-hand (496 ms) responses for smaller numbers (e.g., –8 and –9), [*F*(1,31) = 6.382, *p* < 0.05] (see Figure 2).

**FIGURE 3:**
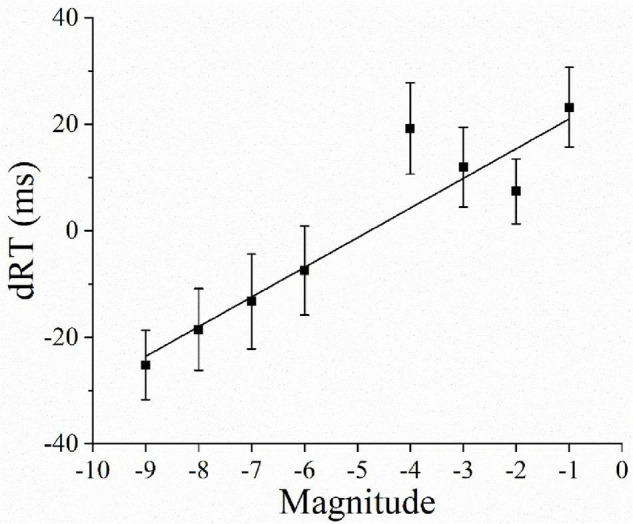
The observed data and regression line represent the RT differences (dRT = right hand RT minus left hand RT) between right-hand and left-hand responses as a function of the magnitudes. Error bars represent 1 SEM.

A one-sample *t*-test revealed that the average regression coefficient was 5.804 and differed significantly from 0 [*B* = 28.694; *t*(31) = 5.866, *p* < 0.001]. This confirmed the presence of a reversed SNARC effect (see Figure 3).

### Discussion

In Experiment 1, we used the magnitude comparison task, in which both the magnitude and spatial-direction were explicitly coded, to explore the spatial representation of negative numbers. The results show a reversed SNARC effect. In other word, small negative numbers are associated with the right, and large negative numbers are associated with the left. That means that small negative numbers are represented on the right, and large negative numbers are represented on the left. Thus, the absolute value of negative numbers associates with space. This is consistent with the findings of Shaki and Petrusic (2005), who used a magnitude comparison task. In their experiment, the sample includes 22 university students who do not have the average age information and the gender information. Our experiment has 32 undergraduate students whose average age is 20.17 (including 15 males and 17 females). Considering both samples are university students, it can be concluded that samples are similar. Their experimental apparatus is a 17-inch Pentium III computer, and our experimental apparatus is a 15.6-inch Lenovo laptop. Thus, both apparatuses are portable computers. Their stimuli include eight negative numbers including –9, –8, –7, –6, –3, –2, –1, 0. And our stimuli include eight negative numbers including –9, –8, –7, –6, –4, –3, –2, –1. Therefore, whether stimuli contain number zero is the most prominent change between our study and theirs. In their study and ours, the presentation time of stimuli is 1000 ms. Through the comparation, our study confirms the fact that the absolute value of negative numbers is indeed associated with space when the magnitude and spatial-direction are explicitly coded, and this kind of spatial representation of negative numbers is not influenced by number zero.

After investigating the condition of explicit coding of magnitude and spatial-direction, we pay attention to next condition of implicit magnitude and explicit spatial-direction coding. The classical study about this condition is the study of attentional SNARC effect.

Fischer et al. (2003) used the altered Posner cuing paradigm to explore SNAs and found small number cues facilitated the detection of a target on the left and large number cues facilitated the detection of a target on the right. This indicated an attentional SNARC effect in positive numbers. However, Colling et al. (2020) tried to replicate Fischer’s (2003a) study with a cross-laboratory large sample and did not find an attentional SNARC effect. Some researchers (Pellegrino et al., 2019; Cipora and Nuerk, 2020) claimed that attentional SNARC effect did not really exist in the field of positive numbers, and passive viewing of positive numbers did not cause attention to shift. However, Fischer et al. (2020) proposed that these failed replication studies ignored the processing depth of number cuing, and “*depth of number processing is a likely moderator of the Att-SNARC effect*” (p. 163). This effect was “*stronger only when participants compute and retain number meaning*” (p. 163), no matter the existence of explicit instructions about the magnitude. On these grounds, we speculated that negative numbers’ attentional SNARC effect may occur when the time of cue increases moderately because participants will have enough time to acquire and retain negative numbers’ semantical meaning in this condition. As Pellegrino et al. (2021) did in their studies for investigating attentional SNARC effect of positive numbers, we increased the time of cue from 300 to 500 ms in a cuing task. Except for the increase of cuing time, other experimental details are a replication of Dodd’s (2011) study of the negative numbers’ attentional SNARC effect. Thus, in the second experiment, we will investigate whether negative numbers have a stable standard or reversed attentional SNARC effect when the cuing time increases. And if an attentional SNARC effect is found, whether absolute or signed values of negative numbers associate with space under the condition of implicit magnitude coding and explicit spatial-direction coding.

## Experiment 2

### Methods

#### Participants

Thirty-four right-handed undergraduate students (23 males, 11 females; mean age = 20.09 years, SD = 2.09) voluntarily participated in the experiment. All participants had normal or corrected-to-normal vision and were unaware of the purpose of the experiment. After a counting task described in Experiment 1, we ensured that all participants had a left-to-right counting habit. All the present studies received approval from the relevant ethics committee.

#### Stimuli and Apparatus

The experiment was performed by using E-Prime 2.0 Professional Software as in Experiment 1. The task design was adapted from those of Fischer et al. (2003). The cuing stimuli were negative numbers: –1, –2, –3, –4, –6, –7, –8, and –9 (1.2°), presented in black, size 48 KaiTi font. The target stimuli were squares with the color changed from transparent to black.

#### Procedure

In a quiet room, participants were seated before a 15.6-inch Lenovo laptop screen with a resolution of 1366 × 768 pixels. The distance from their eyes to the screen was approximately 57 cm. During the experiment, participants were instructed to respond by pressing the space bar on a standard computer keyboard.

**FIGURE 4:**
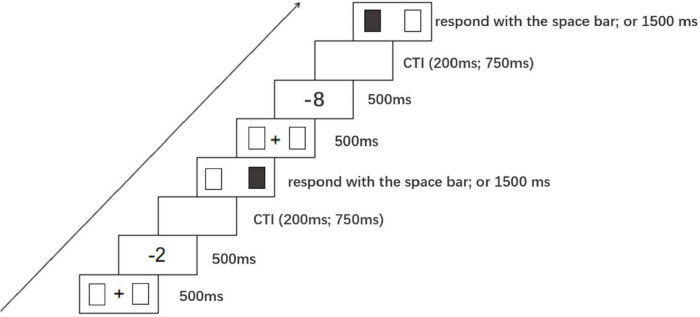
The processing of Experiment 2.

At the beginning of each trial, a central fixation “+” (1.2°) and two blank squares (2° and 4° to the left and right sides of the fixation) were presented on the screen. After 500 ms, the fixation was replaced by a negative number (the cuing stimulus), which was maintained for 500 ms. As previously adopted by Fischer et al. (2003) and by Dodd (2011), the variable cue-target interval (CTI) was either 200 ms (short) or 750 ms (long) to prevent participants from guessing the timing of the sequence. Next, one of the two squares was colored black and participants were required to press the space bar to indicate that they had seen the target. When participants responded with their preferred hand or after 1500 ms elapsed, the squares disappeared, and the trial concluded (see Figure 4). During the formal experiment, there was a total of 320 trials (40 repetitions × 8 negative numbers = 320 trials). Participants were asked to keep their hands close to the space bar, to look at the fixation point in the center of the screen, not to move their eyes for the duration of the experiment, to ignore the magnitude of numbers presented at the fixation point as it was irrelevant to the task, and not to predict the location of the upcoming black square. Prior to the formal experiment, a practice session was run, with all negative numbers presented four times. During the formal experiment, subjects were given a rest period. Cuing stimuli of negative numbers were presented randomly, and colored squares randomly occurred in left or right positions.

### Data Analysis

We used a repeated measures ANOVA with magnitude cues (small = –9 ∼ –6; large = –4 ∼ –1), the position of colored squares (left, right), and the CTI (200 ms, 750 ms) as within-subject factors. The dependent variable was RT.

### Results

**FIGURE 5:**
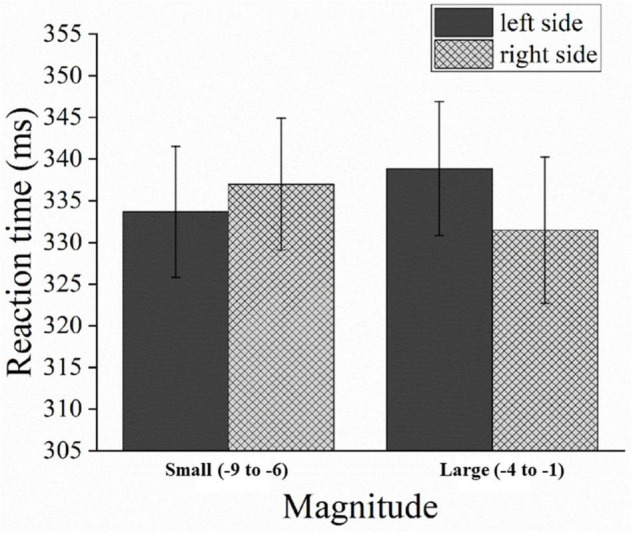
Mean RTs in Experiment 2 as a function of the magnitude cue and the position of colored squares. Error bars represent 1 SEM.

The mean RT was 335 ms (SD = 100 ms). The main effect analysis showed that only CTI had a significant main effect [*F*(1,33) = 134.010, *p* < 0.05, η*^2^_*p*_* = 0.998]. An interaction analysis showed that a significant two-way interaction was observed between the magnitude cue and the position of colored squares [*F*(1,33) = 10.192, *p* < 0.05, η*^2^_*p*_* = 0.505]. The left targets (334 ms) were detected faster than the right targets (337 ms) when smaller negative numbers were used as the cue [*F*(1,33) = 1.797, *p* = 0.189]. The right targets (331 ms) were detected faster than left targets (339 ms) when the magnitude cue was a larger negative number [*F*(1,33) = 6.006, *p* < 0.05] (see Figure 5). Because the two-way interaction effect for the left targets was not significant, and the effect for right targets was significant but small (8 ms). We could not prove a stable attentional SNARC effect just depending on the significant two-way interaction.

Further three-way interaction analysis was conducted and we found a significant three-way interaction of magnitude cue, the position of colored squares, and the CTI [*F*(1,33) = 4.573, *p* < 0.05, η*^2^_*p*_* = 0.203]. For the short CTI, left targets (359 ms) were detected faster than right targets (366 ms) with smaller magnitude cues [*F*(1,33) = 4.770, *p* < 0.05]. Right targets (356 ms) were detected faster than left targets (366 ms) with larger magnitude cues [*F*(1,33) = 5.081, *p* < 0.05]. This indicated the presence of an attentional SNARC effect under the short CTI condition. However, for the long CTI, regardless of larger or smaller magnitude cues, the right targets were always detected faster than the left targets [larger magnitude cues: 306 ms vs. 312 ms, *F* (1, 33) = 2.711, *p* = 0.109; smaller magnitude cues: 307 ms vs. 308 ms, *F*(1,33) = 0.037, *p* = 0.849]. This indicated the lack of a standard or reversed attentional SNARC effect under the long CTI condition.

### Discussion

In Experiment 2, we used the cuing task, in which only the spatial-direction was explicitly coded, to explore the spatial representation of negative numbers. The results showed a stable attentional SNARC effect in short CTI condition but not in long CTI condition. This suggests that when the spatial-direction is explicitly coded and the magnitude is implicitly coded, the SNAs of negative numbers only exist under short CTI conditions. In this condition, the signed value of negative numbers associates with space. This result is inconsistent with those of Dodd (2011). In their study, they didn’t find any attentional or reversed attentional SNARC effect despite short or long stimulus onset asynchrony. The potential reason for the difference may derive from the cuing time. In Dodd’s (2011) study, the time is 300 ms, which is not enough for participants to establish an obligatorily semantical processing about the magnitude of negative numbers. However, in our study, we increase the time to 500ms, consequently find an attentional SNARC effect. Comparing the two studies, except for the difference of cuing time, other experimental details are similar. In their study, the sample includes 37 undergraduate students who do not have the average age information and the gender information. In our study, 34 undergraduate students whose average age is 20.09 (including 23 males and 11 females) participate in our experiment. Considering both samples are undergraduate students, it can be concluded that samples are similar. Their experimental apparatus is a Pentium IV PC, and our experimental apparatus is a 15.6-inch Lenovo laptop. Thus, both apparatuses are general-purpose computers. Their cuing stimuli are pure negative numbers, including –1, –2, –8, –9, and our cuing stimuli are pure negative numbers too, including -1, -2, -3, -4, -6, -7 -8, -9. Both their target stimuli and our target stimuli are squares. The only difference between Dodd’s (2011) and our studies is the cuing time. Thus, we conclude that the attentional SNARC effect of pure negative numbers only exists under a condition of a relatively longer cuing time (for acquiring negative numbers’ semantical meaning) as well as a relatively shorter CTI (for retaining negative numbers’ semantical meaning). Recently, this possibility has been proposed by discoverers of the attentional SNARC effect, although they haven’t proved it by carrying out an empirical study (Fischer et al., 2020). In addition, our result is consistent with Fischer’s (2003) study, in which mixed positive and negative numbers were used as cues in a cuing task. Results indicated that left-hand responses were faster when the stimulus was a negative number, while the right-hand responses were faster when the stimulus was a positive number, revealing an attentional SNARC effect. Thus, although the two studies of Fischer’s and ours adopted different number stimuli, both studies have indicated the presence of an attentional SNARC effect. Namely, if there are SNAs for negative numbers, the signed value of negative numbers is associated with space when participants implicitly code the magnitude and explicitly code the spatial-direction.

After investigating the condition of implicit magnitude and explicit spatial-direction, we pay attention to next condition of explicit magnitude and implicit spatial-direction. The classical study about this condition adopts the magnitude and picture-direction classification tasks with Go/No-Go responses. Initially, this task is invented by Fischer and Shaki (2016) to prove the conceptual association between positive numbers and space. Later, this task is used in the field of negative numbers to explore the SNARC effect of negative numbers. The classical study in the field is coming from Fischer and Shaki (2017). In their study, participants were required to press the space bar in 50% of trials with their right hand. Therefore, the spatial-direction is implicitly coded during number assessment. They observed that for left-to-right counters, there was a SNARC effect. However, for right-to-left counters, stable SNAs disappeared. Their results proved that individuals’ spatial representation of negative numbers depended on individuals’ counting habits.

However, in their task, the stimuli of car pictures include mixed directivity information. The mixed directivity information comes from the directivity of pictures and the directivity of driving rules. Therefore, in our third experiment, we repeated and improved the experiment of Fischer and Shaki (2017) by replacing clipboard pictures of cars with more abstract arrow symbols to exclude the contamination of the directivity of driving rules. Namely, we adopt the magnitude and arrow-direction classification task with Go/No-Go responses. And we can accurately explore whether negative numbers have a standard or reversed SNARC effect under the condition of explicit magnitude and implicit spatial-direction.

## Experiment 3

### Methods

#### Participants

Thirty-one right-handed undergraduate students (21 males, 10 females; mean age = 19.81 years, SD = 1.35) voluntarily participated in the experiment. All participants had normal or corrected-to-normal vision and were unaware of the purpose of the experiment. After a counting task described in Experiment 1, we ensured that all participants had a left-to-right counting habit. The present studies received approval from the relevant ethics committee.

#### Stimuli and Apparatus

The experiment was performed by using E-Prime 2.0 Professional Software as in Experiment 1. The experimental task was adapted from that of Fischer and Shaki (2017). The target stimuli were (1) negative numbers (–1, –2, –3, –4, –6, –7, –8, and –9) presented in black, 48 size KaiTi font; and (2) two arrow pictures (pointing left or right; size 2.2 × 1.2 cm).

#### Procedure

In a quiet room, participants were seated before a 15.6-inch Lenovo laptop screen with a resolution of 1366 × 768 pixels. The distance from their eyes to the screen was approximately 57 cm. During the experiment, participants were instructed to focus on the number magnitude or the arrow orientation, and only to respond to numbers or arrows fitting for the responsive instructions. Participants responded by pressing the space bar on a standard computer keyboard.

This task comprised four separate blocks corresponding with four different responsive instructions. Block1: “respond only to numbers smaller than minus five or left-facing arrow”; block2: “respond only to numbers larger than minus five or right-facing arrow”; block3: “respond only to numbers larger than minus five or left-facing arrow”; block4: “respond only to numbers smaller than minus five or right-facing arrow.”

According to the definition of SNARC effect, small numbers associate with the left space, and large numbers associate with the right space. Thus, the responsive instruction with a combination of small numbers and left-facing arrow (or the responsive instruction with a combination of large numbers and right-facing arrow) is congruent with the SNARC effect. Conversely, the responsive instruction with a combination of large numbers and left-facing arrow (or the responsive instruction with a combination of small numbers and right-facing arrow) is incongruent with the SNARC effect. The congruence (whether the responsive instruction is congruent or incongruent with the SNARC effect) is the logic of our following data analysis.

In each block, there were two sessions, namely practice sessions and formal sessions. In the practice session, each negative number was presented one time and each arrow picture was presented four times. In the formal session, eight negative numbers were repeated five times, and two arrow pictures were repeated twenty times. Hence, for this experiment, there were 64 trials [(8 negative numbers + 4 repetitions × 2 arrows) × 4 blocks = 64 trials] in practice sessions, and 320 trials [(5 repetitions × 8 negative numbers + 20 repetitions × 2 arrows) × 4 blocks = 320 trials] in formal sessions.

**FIGURE 6:**
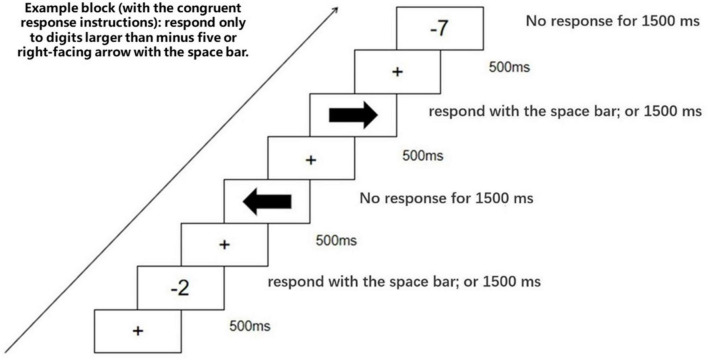
The processing of Experiment 3.

At the beginning of each block, the responsive instructions were displayed until participants read and understood them. Next, a fixation (“+”) was displayed in the center of the screen against a white background. After 500 ms, the fixation was replaced by a negative number or an arrow picture (both the target stimuli), which disappeared after 1500 ms or after a response made by participants. The trial then concluded (see Figure 6). In sum, participants respond in 50% of the trials because they only respond to numbers or arrows fitting for the responsive instructions, and stimuli match with the responsive instructions in 50% of the trials. The block order was counterbalanced across participants. And stimuli were presented in a random order.

### Data Analysis

For simplifying the analysis, as Fischer and Shaki (2016) did, we defined the block1 and block2 as congruent-responsive instructions block, and defined block3 and block4 as incongruent-responsive instructions block. Thus, we manufactured another factor, which was defined as the responsive congruence (including two levels: congruent, incongruent). Data from the block1 and block2 were merged, and then the merged data from block1 and block2 composed the data of the congruent level. The same operation was conducted for the data of block3 and block4. Consequently, the merged data from block3 and block4 composed the data of the incongruent level. By adopting this approach, left- and right-located response keys can be excluded, thereby ensuring an implicit coding of spatial-direction. For statistical results, a significant main effect of the responsive congruence is equivalent to a significant interaction effect between the magnitude and the space (space are oriented by arrows’ direction), and indicates the existence of a SNARC or reversed SNARC effect.

According to the statistical approach from Fischer and Shaki (2016), we used a repeated measures ANOVA with the magnitude (small “<–5”, large “>–5”) and the responsive congruence (congruent, incongruent) as within-subject factors. The dependent variable was RT.

### Results

**FIGURE 7:**
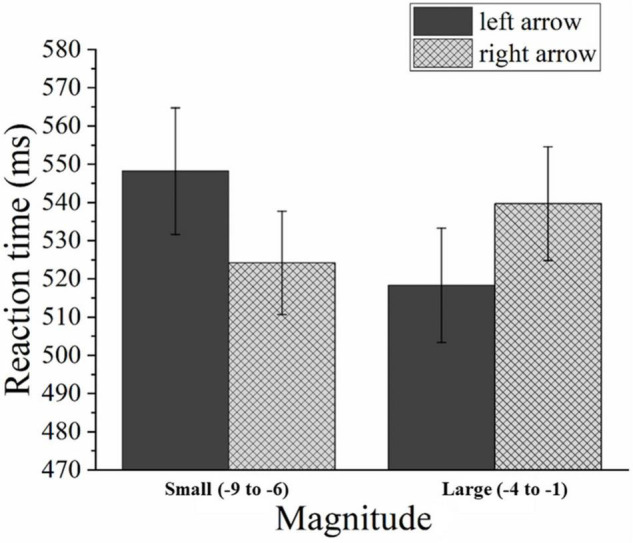
Mean RTs in Experiment 3 as a function of magnitude and arrow orientation. Error bars represent 1 SEM.

The mean error rate was 0.66%, including 0.06% commission errors and 0.6% omission errors. Trials in which an error occurred were excluded from the analysis. The mean RT for correct trials was 533 ms (SD = 138 ms). The responsive congruence had a significant main effect [*F*(1,30) = 11.337, *p* < 0.05, η*^2^_*p*_* = 0.511]. Through further analysis, we found that responses to smaller numbers (smaller than minus five) were faster when the arrow faced the right (524 ms) (in block 4) compared to the left (548 ms) (in block 1), [*F*(1,30) = 6.037, *p* < 0.05]. Moreover, responses to larger numbers (larger than minus five) were faster when the arrow faced the left (518 ms) (in block 3) compared to the right (540 ms) (in block 2), [*F*(1,30) = 5.709, *p* < 0.05] (see Figure 7). This demonstrates a reversed SNARC effect. Namely, the absolute value of negative numbers associates with space.

### Discussion

In Experiment 3, we used the magnitude and arrow-direction classification task with Go/No-Go responses. In the task, although participants are required to focus on arrow directions, the spatial-direction is implicitly coded according to its definitions. The results showed that responses to smaller numbers were faster when the arrow faced the right, and responses to larger numbers were faster when the arrow faced the left. This suggests that the absolute value of negative numbers associates with space in this condition. Combining the result of the first and second experiments, the results of Experiment 3 also proved that the association between negative numbers and space are not triggered by the utilizing of left- and right located response keys. Comparing with the previous studies, it can be found that our results are inconsistent with the result of Fischer and Shaki (2017). In their study, participants with a left-to-right counting habit have a SNARC effect of negative numbers, which means that singed values of negative numbers are associated with space. However, participants with a right-to-left counting habit do not have stable SNAs of negative numbers. In our study, we recruit Chinese university students as our participants. They all have a left-to-right counting habit as well as a common sense of driving along the right side of the road. We obtain a reversed SNARC effect of negative numbers exists for individuals who have a left-to-right counting habit. Comparing the study of Fischer and Shaki (2017) with the study of ours, except for the difference of stimuli, other experimental details are similar. In their study, the sample includes 51 students whose average age is 22.50 (including 5 males and 46 females). In our study, 31 undergraduate students whose average age is 19.81 (including 21 males and 10 females) participate in our experiment. Considering both samples are adult students with similar ages, it can be concluded that samples are alike. Their experimental apparatus is a 19-inch PC monitor, and our experimental apparatus is a 15.6-inch Lenovo laptop. Thus, both apparatuses are general-purpose computers. Their stimuli are pure negative numbers (including –1, –2, –8, –9) and four clipboard pictures of left-facing (or right-facing) cars. However, our cuing stimuli are pure negative numbers (including –1, –2, –3, –4, –6, –7 –8, –9) and two arrow pictures. Therefore, their task is the magnitude and picture-direction classification task with Go/No-Go responses, whereas our task is the magnitude and arrow-direction classification task with Go/No-Go responses. To sum up, because we adopt a more rigorous task, eliminating the interference of the mixed directivity information contained in driving rules, we acquire a more reliable result. That can be the potential reason leading to the difference between our results and those of Fischer and Shaki (2017). Currently, we prove that negative numbers have a reversed SNARC effect under the condition of an explicit magnitude as well as an implicit spatial-direction for participants with left-to-right counting habits. Namely, the absolute value of negative numbers is associated with space in this condition. Moreover, the findings of our Experiment 1 and Experiment 3 indicate that when the magnitude is explicitly coded, the absolute value of negative numbers is associated with space despite the coding level of spatial-direction, at least, for individuals with left-to-right counting habits.

## General Discussion

Negative numbers consist of a minus sign and magnitude. According to the fundamental rule of mathematics, negative numbers have absolute values and signed values. The current study aims to determine whether negative numbers have a SNARC effect (or a reversed SNARC effect), and which types of values are associated with space to elucidate the spatial representation of negative numbers. In addition, we intended to find modulating factors of the spatial representation of negative numbers.

Shaki and Fischer (2018) examined the SNARC effect of positive numbers and proposed that different tasks included distinct coding levels of the magnitude and spatial-direction, which may lead to inconformity of numbers’ spatial representation. To investigate whether the coding level of magnitude and spatial-direction is a modulating factor for spatial representation of negative numbers, we manipulated the coding level of the magnitude and spatial-direction by replicating three classical studies which fitting for three combinations of coding levels.

Through replicating and improving these classical studies, it is proved that negative numbers have a flexible spatial representation. The coding level of the magnitude and spatial-direction can influence the spatial representation of negative numbers and change the type of values associating with space. The spatial representation varies with the change of coding levels of magnitude and spatial-direction. When the magnitude and spatial-direction are explicitly coded in Experiment 1, the absolute value of negative numbers is associated with space. When the magnitude is explicitly coded and the spatial-direction is implicitly coded in Experiment 3, the absolute value of negative numbers is associated with space. Contrarily, when the magnitude is implicitly coded and the spatial-direction is explicitly coded in Experiment 2, the signed value of negative numbers is associated with the space. To summarize, negative numbers have a reversed SNARC effect when the magnitude of negative numbers is explicitly coded, no matter employing the explicit or implicit spatial-direction; negative numbers have an attentional SNARC effect under the condition of implicit magnitude as well as explicit spatial-direction (the effect only exists in the short CTI) (see Table 2).

**TABLE 2:** Summary of results for each combination of coding levels in different experiments of this study (for details, see text).

Magnitude		Explicit	Implicit
Spatial-direction	Explicit	See in Experiment 1: • A reversed SNARC effect exists. The absolute value of negative numbers is associated with space.	See in Experiment 2: • An attentional SNARC effect exists under the short CTI condition. The signed value of negative numbers is associated with space. • An attentional SNARC effect does not exist under the long CTI condition.
	Implicit	See in Experiment 3: • A reversed SNARC effect exists. The absolute value of negative numbers is associated with space.	• None

A possible explanation for this phenomenon is that numbers from 1 to 9 are numeric primitives (Tzelgov et al., 2009), and other numbers (such as negative numbers, fractions, decimals, and double digits) are evolved from these numeric primitives. Thus, when the magnitude is explicitly coded, the spatial representation of negative numbers is susceptible to numeric primitives. This triggers the representation of the absolute value, which is equal to the value of numeric primitive, on the MNL. However, if the spatial-direction is explicitly coded with the implicit magnitude, the representation of negative numbers is not strongly affected by numeric primitives. Rather, it is affected by mathematical learning experiences (e.g., –1 and –2 are defined as larger numbers, and –8 and –9 are defined as smaller numbers). The reason is as follows. Because of the absence of explicit magnitude, the influence of magnitude on participants becomes faint. At the same time, the implicit magnitude still leads to an obligatorily semantic processing of numbers, if the coding time is enough for participants to acquire and retain the semantic meaning of negative numbers. Thus, participants can obtain the mathematical definition of numbers. And the signed value of negative numbers is represented on the MNL.

Our findings also resonate with the model proposed by Prpic et al. (2016) in their study of the SNARC-like effects in musical note values. In their model, the authors “*hypothesize the existence of two separate mechanisms underlying the SNARC-like effects: a more general Order-Related Mechanism (ORM) and a specific Magnitude-Related Mechanism (MRM)*” (p. 1248). Specifically, when participants indirectly operate the magnitude of stimuli, the ORM is a general mechanism to support the SNARC-like effects. In this condition, the stimuli are processed in the same way independently of their semantic information. At least, “*in western culture, this spatial representation is preferentially oriented from left-to-right*” (Prpic et al., 2016, p. 1248). However, when a task provides participants with meaningful information about magnitude of the stimuli, both MRM and ORM can be activated. Moreover, if the order and magnitude of stimuli have conflicts, the MRM prevails, and stimuli are processed relying on their semantic meaning.

For negative numbers, there is an undisputed conflict between their order and magnitude. Their order is in accordance with absolute values, while their magnitude is in accordance with signed values. According to the model of Prpic et al. (2016, 2021), the reversed SNARC effect of negative numbers in our Experiment 1 and Experiment 3 can be explained by MRM because the magnitude of negative numbers is explicitly coded. Namely, participants are instructed to focus on the magnitude in our Experiment 1 and Experiment 3, which provides participants with meaningful information about magnitude and leads participants to represent the absolute value of negative numbers in a left to right order (–1 on the left, –9 on the right). At the same time, the attentional SNARC effect of negative numbers in our Experiment 2 also can be interpreted by ORM due to the implicit magnitude and explicit spatial-direction. Namely, participants are required to ignore the magnitude in our Experiment 2, which triggers participants representing the signed values (semantic values) of negative numbers in a left to right MNL (–9 on the left, –1 on the right).

## Conclusion

The current study confirms the existence of a flexible SNAs of negative numbers and elucidates two modulating factors on negative numbers’ spatial representing in the mind. The two modulating factors are magnitude and spatial-direction. The spatial representation of negative numbers changes with the joint coding level of the two modulating factors. Thus, negative numbers have SNARC effect as well as the reversed SNARC effect. In different combinations of coding level, the spatial representation of negative numbers is different.

However, there are also some limitations of the current study. First, the participants are mainly young and healthy university students, and all of them have left-to-right counting habits. It remains to be explored whether our results are applicable to older or younger subjects and are applicable to individuals with a right-to-left counting habit. Second, the result of our second experiment is data-driven to some extent. We acknowledge that it has not substantially reduced the uncertainty around the existence of the attentional SNARC effect. Further studies should examine our conclusions by adopting various possible cuing times. Third, our study just explores three combinations of coding levels of magnitude and spatial-direction. The condition about the implicit coding level of magnitude as well as spatial-direction is not systematically explored. Although in this condition, according to the view of Shaki and Fischer (2018), there is a high probability that the SNAs of negative numbers does not exist, it is still valuable to prove it by empirical research.

## Data Availability Statement

The raw data supporting the conclusions of this article will be made available by the authors, without undue reservation.

## Ethics Statement

The studies involving human participants were reviewed and approved by Psychology Research Ethics Review Committee, School of Psychology, Guizhou Normal University. The patients/participants provided their written informed consent to participate in this study.

## Author Contributions

XZ: writing introduction and designing experiments. JZ: writing original draft preparation and co-designing experiments. LD: executing experiments and data analysis. YP: conceptualization, data curation, supervision, and editing. All authors contributed to the article and approved the submitted version.

## Conflict of Interest

The authors declare that the research was conducted in the absence of any commercial or financial relationships that could be construed as a potential conflict of interest.

## Publisher’s Note

All claims expressed in this article are solely those of the authors and do not necessarily represent those of their affiliated organizations, or those of the publisher, the editors and the reviewers. Any product that may be evaluated in this article, or claim that may be made by its manufacturer, is not guaranteed or endorsed by the publisher.

## References

[B1] Aleotti S. Di Girolamo F. Massaccesi S. Priftis K. (2020). Numbers around Descartes: a preregistered study on the three-dimensional SNARC effect. *Cognition* 195:104111. 10.1016/j.cognition.2019.104111 31731115

[B2] Antoine S. Gevers W. (2016). Beyond left and right: automaticity and flexibility of number-space associations. *Psychon. Bull. Rev.* 23 148–155. 10.3758/s13423-015-0856-x 25968089

[B3] Cipora K. Nuerk H. C. (2020). *What the Attentional-SNARC and its (null) Replications Can and Cannot Tell Us.* Available online at: https://psyarxiv.com/a5k3h (accessed May 01, 2022).

[B4] Cleland A. A. Bull R. (2019). Automaticity of access to numerical magnitude and its spatial associations: the role of task and number representation. *J. Exp. Psychol.* 45 333–348. 10.1037/xlm0000590 29708369

[B5] Colling L. J. Szűcs D. De Marco D. Cipora K. Ulrich R. Nuerk H. (2020). A multilab registered replication of the attentional SNARC effect. *Adv. Methods Pract. Psychol. Sci.* 3 143–162.

[B6] Dehaene S. Bossini S. Giraux P. (1993). The mental representation of parity and number magnitude. *J. Exp. Psychol.* 122 371–396. 10.1037/0096-3445.122.3.371

[B7] Didino D. Breil C. Knops A. (2019). The influence of semantic processing and response latency on the SNARC effect. *Acta Psychol.* 196 75–86. 10.1016/j.actpsy.2019.04.008 31004938

[B8] Dixon P. (2017). Episodic retrieval and the SNARC effect. *Psychon. Bull. Rev.* 24 1943–1948. 10.3758/s13423-017-1253-4 28244017

[B9] Dodd M. D. (2011). Negative numbers eliminate, but do not reverse, the attentional SNARC effect. *Psychol. Res.* 75 2–9. 10.1007/s00426-010-0283-6 20379741

[B10] Fischer M. H. (2003). Cognitive Representation of Negative Numbers. *Psychol. Sci.* 14 278–282. 10.1111/1467-9280.03435 12741754

[B11] Fischer M. H. Castel A. D. Dodd M. D. Pratt J. (2003). Perceiving numbers causes spatial shifts of attention. *Nat. Neurosci.* 6 555–556. 10.1038/nn1066 12754517

[B12] Fischer M. H. Dodd M. D. Castel A. D. Pratt J. (2020). The Unbearable Lightness of Attentional Cuing by Symbolic Magnitude: commentary on the Registered Replication Report by Colling et al. *Adv. Methods Pract. Psychol. Sci.* 3 163–165. 10.1177/2515245920902743

[B13] Fischer M. H. Roitmann J. (2005). Do negative numbers have a place on the mental number line. *Psychol. Sci.* 47 22–32.

[B14] Fischer M. H. Shaki S. (2016). Measuring spatial–numerical associations: evidence for a purely conceptual link. *Psychol. Res.* 80 109–112. 10.1007/s00426-015-0646-0 25617061

[B15] Fischer M. H. Shaki S. (2017). Implicit spatial-numerical associations: negative numbers and the role of counting direction. *J. Exp. Psychol.* 43 639–643. 10.1037/xhp0000369 28345942

[B16] Gertner L. Henik A. Reznik D. Cohen Kadosh R. (2013). Implications of number-space synesthesia on the automaticity of numerical processing. *Cortex* 49 1352–1362. 10.1016/j.cortex.2012.03.019 22578710 PMC3428851

[B17] Gevers W. Verguts T. Reynvoet B. Caessens B. Fias W. (2006). Numbers and space: a computational model of the SNARC effect. *J. Exp. Psychol.* 32 32–44. 10.1037/0096-1523.32.1.32 16478324

[B18] Kopiske K. K. Löwenkamp C. Eloka O. Schiller F. Kao C. S. Wu C. (2016). The SNARC effect in Chinese numerals: do visual properties of characters and hand signs influence number processing? *PLoS One* 11:e0163897. 10.1371/journal.pone.0163897 27684956 PMC5042428

[B19] Lorch R. F. Myers J. L. (1990). Regression analyses of repeated measures data in cognitive research. *J. Exp. Psychol.* 16 149–157. 10.1037/0278-7393.16.1.149 2136750

[B20] Mingolo S. Prpic V. Bilotta E. Fantoni C. Agostini T. Murgia M. (2021). Snarcing with a phone: the role of order in spatial-numerical associations is revealed by context and task demands. *J. Exp. Psychol.* 47 1365–1377. 10.1037/xhp0000947 34766820

[B21] Nan W. Yan L. Yang G. Liu X. Fu S. (2021). Two processing stages of the SNARC effect. *Psychol. Res.* 86 375–385. 10.1007/s00426-021-01506-5 33847782

[B22] Nuerk H. Iversen W. Willmes K. (2004). Notational Modulation of the SNARC and the MARC (Linguistic Markedness of Response Codes) Effect. *Q. J. Exp. Psychol. Sec. A* 57 835–863. 10.1080/02724980343000512 15204120

[B23] Pellegrino M. Pinto M. Marson F. Lasaponara S. Doricchi F. (2021). Perceiving numerosity does not cause automatic shifts of spatial attention. *Exp. Brain Res.* 239 3023–3034. 10.1007/s00221-021-06185-7 34355249 PMC8536601

[B24] Pellegrino M. Pinto M. Marson F. Lasaponara S. Rossi-Arnaud C. Cestari V. (2019). The Attentional-SNARC effect 16 years later: no automatic space–number association (taking into account finger counting style, imagery vividness, and learning style in 174 participants). *Exp. Brain Res.* 237 2633–2643. 10.1007/s00221-019-05617-9 31384968

[B25] Pinto M. Pellegrino M. Marson F. Lasaponara S. Doricchi F. (2019). Reconstructing the origins of the space-number association: spatial and number-magnitude codes must be used jointly to elicit spatially organised mental number lines. *Cognition* 190 143–156. 10.1016/j.cognition.2019.04.032 31079015

[B26] Pitt B. Casasanto D. (2020). The correlations in experience principle: how culture shapes concepts of time and number. *J. Exp. Psychol.* 149 1048–1070. 10.1037/xge0000696 31633369

[B27] Prpic V. Fumarola A. De Tommaso M. Luccio R. Murgia M. Agostini T. (2016). Separate mechanisms for magnitude and order processing in the spatial-numerical association of response codes (SNARC) effect: the strange case of musical note values. *J. Exp. Psychol.* 42 1241–1251. 10.1037/xhp0000217 26950384

[B28] Prpic V. Mingolo S. Agostini T. Murgia M. (2021). Magnitude and order are both relevant in SNARC and SNARC-like effects: a commentary to Casasanto & Pitt (2019). *Cogn. Sci.* 45:e13006. 10.1111/cogs.13006 34213789

[B29] Psychology Software Tools Inc (2017). *E-Prime: Documentation Article.* Pennsylvania: Psychology Software Tools Inc.

[B30] Shaki S. Fischer M. H. (2018). Deconstructing spatial-numerical associations. *Cognition* 175 109–113. 10.1016/j.cognition.2018.02.022 29500963

[B31] Shaki S. Petrusic W. M. (2005). On the mental representation of negative numbers: context-dependent SNARC effects with comparative judgments. *Psychon. Bull. Rev.* 12 931–937. 10.3758/BF03196788 16524013

[B32] Shan W. Ma W. Yu N. (2017). “Dose the Difference in Driving Rules Matter for Security ?,” in *International Conference on Management Science and Engineering (ICMSE).* (Japan: IEEE), 219–227.

[B33] Tzelgov J. Ganor-Stern D. Maymon-Schreiber K. (2009). The Representation of Negative Numbers: exploring the Effects of Mode of Processing and Notation. *Q. J. Exp. Psychol.* 62 605–624. 10.1080/17470210802034751 18609405

[B34] Varma S. Schwartz D. L. (2011). The mental representation of integers: an abstract-to-concrete shift in the understanding of mathematical concepts. *Cognition* 121 363–385. 10.1016/j.cognition.2011.08.005 21939966

[B35] Wood G. Nuerk H. C. Willmes K. (2006). Crossed Hands and the Snarc Effect: afailure to Replicate Dehaene, Bossini and Giraux (1993). *Cortex* 42 1069–1079. 10.1016/S0010-9452(08)70219-3 17209413

[B36] Yan L. Yang G. Nan W. Liu X. Fu S. (2021). The SNARC effect occurs in the response-selection stage. *Acta Psychol.* 215:103292. 10.1016/j.actpsy.2021.103292 33740617

